# Supplementary material for: Introduction, Dispersal, and Predominance of SARS-CoV-2 Delta Variant in Rio Grande do Sul, Brazil: A Retrospective Analysis
Source: Microorganisms. 2023 Dec 7;11(12):2938. doi: 10.3390/microorganisms11122938 (PMC10745878; doi:10.3390/microorganisms11122938)
Supplement: Supplementary file 1 [file microorganisms-11-02938-s001.zip › Table S4.docx]

Table S4. Mutational profile of VOC Gamma

| **Name** | **Polymorphism Type** | **Minimum** | **Maximum** | **Locus** | **Change** | **Códon change** | **AA change** | **Variant Frequency** | **Strand-Bias >65% P-value** |
| --- | --- | --- | --- | --- | --- | --- | --- | --- | --- |
| T | SNP (transversion) | 1 | 1 | 5'-UTR | A -> T | - | - | 25.0% | 1.0 |
| T | SNP (transition) | 241 | 241 | 5'-UTR | C -> T | - | - | 100.0% | 1.3E-49 |
| C | SNP (transition) | 733 | 733 | ORF1ab | T -> C | GAT -> GAC | - | 99.6% | 1.9E-49 |
| T | SNP (transition) | 2,749 | 2,749 | ORF1ab | C -> T | GAC -> GAT | - | 96.6% | 6.0E-48 |
| T | SNP (transition) | 3,037 | 3,037 | ORF1ab | C -> T | TTC -> TTT | - | 100.0% | 1.3E-49 |
| T | SNP (transition) | 3,828 | 3,828 | ORF1ab | C -> T | TCA -> TTA | S1188L | 99.2% | 3.0E-49 |
| C | SNP (transversion) | 5,648 | 5,648 | ORF1ab | A -> C | AAA -> CAA | K1795Q | 100.0% | 1.3E-49 |
| G | SNP (transition) | 6,319 | 6,319 | ORF1ab | A -> G | CAA -> CCG | - | 100.0% | 3.0E-49 |
| G | SNP (transition) | 6,613 | 6,613 | ORF1ab | A -> G | GTA -> GTG | - | 100.0% | 1.3E-49 |
|  | Deletion | 11,28 | 11,288 | ORF1ab | #NOME? | - | - | 99.6% | 1.7E-48 |
| A | SNP (transition) | 11,291 | 11,291 | ORF1ab | G -> A | GGT -> AGT | G3676S | 99.2% | 2.6E-48 |
| G | SNP (transversion) | 11,296 | 11,296 | ORF1ab | T -> G | TTT -> TTG | F3677L | 99.2% | 1.7E-48 |
| T | SNP (transition) | 12,778 | 12,778 | ORF1ab | C -> T | TAC -> TAT | - | 96.6% | 6.0E-48 |
| T | SNP (transition) | 13,86 | 13,86 | ORF1ab | C -> T | GAC -> GAT | - | 96.6% | 6.0E-48 |
| T | SNP (transition) | 14,408 | 14,408 | ORF1ab | C -> T | CCT -> CTT | P4714L | 100.0% | 1.3E-49 |
| T | SNP (transversion) | 17,259 | 17,259 | ORF1ab | G -> T | GAG -> GAT | E5664D | 96.6% | 6.0E-48 |
| T | SNP (transition) | 21,614 | 21,614 | S | C -> T | CTT -> TTT | L18F | 100.0% | 1.3E-49 |
| A | SNP (transversion) | 21,621 | 21,621 | S | C -> A | ACC -> AAC | T20N | 96.6% | 6.0E-48 |
| T | SNP (transition) | 21,638 | 21,638 | S | C -> T | CCT -> TCT | P26S | 100.0% | 1.3E-49 |
| T | SNP (transversion) | 21,974 | 21,974 | S | G -> T | GAT -> TAT | D138Y | 100.0% | 1.3E-49 |
| T | SNP (transversion) | 22,132 | 22,132 | S | G -> T | AGG -> AGT | N188S | 99.2% | 1.2E-46 |
| C | SNP (transversion) | 22,812 | 22,812 | S | A -> C | AAG -> ACG | K417T | 98.5% | 7.0E-49 |
| A | SNP (transition) | 23,012 | 23,012 | S | G -> A | GAA -> AAA | E484K | 100.0% | 8.0E-47 |
| T | SNP (transversion) | 23,063 | 23,063 | S | A -> T | AAT -> TAT | N501Y | 100.0% | 5.2E-47 |
| G | SNP (transition) | 23,403 | 23,403 | S | A -> G | GAT -> GGT | D614G | 100.0% | 1.3E-49 |
| T | SNP (transition) | 23,525 | 23,525 | S | C -> T | CAT -> TAT | H655Y | 100.0% | 1.7E-48 |
| T | SNP (transition) | 24,642 | 24,642 | S | C -> T | ACT -> ATT | T1027I | 100.0% | 1.3E-49 |
| T | SNP (transversion) | 25,088 | 25,088 | S | G -> T | GTT -> TTT | V1176F | 100.0% | 1.9E-49 |
| C | SNP (transition) | 26,149 | 26,149 | ORF3a | T -> C | TCC -> CCC | S253P | 99.2% | 7.0E-49 |
| A | SNP (transition) | 28,167 | 28,167 | ORF8 | G -> A | GAA -> AAA | E92K | 96.1% | 8.0E-47 |
| AACA | Insertion | 28,263 | 28,262 | Intergenic | #NOME? | - | - | 98.5% | 7.0E-49 |
| G | SNP (transversion) | 28,512 | 28,512 | N | C -> G | CCA -> CGA | P80R | 100.0% | 4.6E-49 |
| TC | Substitution | 28,877 | 28,878 | N | AG -> TC | - | - | 100.0% | 3.0E-49 |
| AAC | Substitution | 28,881 | 28,883 | N | GGG -> AAC | - | - | 100.0% | 3.0E-49 |
| A | SNP (transversion) | 29,834 | 29,834 | 3'-UTR | T -> A | - | - | 89.7% | 2.2E-18 |
| A | SNP (transversion) | 29,858 | 29,858 | 3'-UTR | T -> A | - | - | 70.0% | 0.098 |
| A | SNP (transversion) | 29,867 | 29,867 | 3'-UTR | T -> A | - | - | 100.0% | 0.85 |
