# Supplementary material for: Introduction, Dispersal, and Predominance of SARS-CoV-2 Delta Variant in Rio Grande do Sul, Brazil: A Retrospective Analysis
Source: Microorganisms. 2023 Dec 7;11(12):2938. doi: 10.3390/microorganisms11122938 (PMC10745878; doi:10.3390/microorganisms11122938)
Supplement: Supplementary file 1 [file microorganisms-11-02938-s001.zip › Table S5.docx]

Table S5. Mutational profile of VOC Delta

| **Name** | **Polymorphism Type** | **Minimum** | **Maximum** | **Locus** | **Change** | **Códon change** | **AA change** | **Variant Frequency** | **Strand-Bias >65% P-value** |
| --- | --- | --- | --- | --- | --- | --- | --- | --- | --- |
| T | SNP (transversion) | 210 | 210 | 5'-UTR | G -> T | - | - | 100.0% | 1.5E-91 |
| T | SNP (transition) | 241 | 241 | 5'-UTR | C -> T | - | - | 100.0% | 4.3E-92 |
| T | SNP (transition) | 3,037 | 3,037 | ORF1ab | C -> T | TTC -> TTT | - | 100.0% | 7.6E-93 |
| G | SNP (transition) | 3,536 | 3,536 | ORF1ab | A -> G | ATA -> GAT | I1091D | 68.6% | 1.2E-63 |
| T | SNP (transversion) | 4,181 | 4,181 | ORF1ab | G -> T | GCT -> TCT | A1306S | 99.4% | 2.8E-92 |
| T | SNP (transition) | 4,927 | 4,927 | ORF1ab | C -> T | GAC -> GAT | - | 68.6% | 1.8E-63 |
| G | SNP (transition) | 5,638 | 5,638 | ORF1ab | A -> G | AAA -> AAG | - | 68.6% | 1.2E-63 |
| T | SNP (transition) | 6,402 | 6,402 | ORF1ab | C -> T | CCA -> CTA | P2046L | 99.4% | 4.3E-92 |
| T | SNP (transition) | 7,124 | 7,124 | ORF1ab | C -> T | CCT -> TCT | P2287S | 99.4% | 2.8E-92 |
| T | SNP (transition) | 8,986 | 8,986 | ORF1ab | C -> T | GAC -> GAT | - | 99.4% | 2.0E-87 |
| T | SNP (transversion) | 9,053 | 9,053 | ORF1ab | G -> T | GTA -> TTA | V2930L | 99.4% | 2.3E-88 |
| T | SNP (transition) | 10,029 | 10,029 | ORF1ab | C -> T | ACC -> ATC | T3255I | 99.4% | 2.8E-92 |
| G | SNP (transition) | 11,201 | 11,201 | ORF1ab | A -> G | ACT -> GCT | T3646A | 99.4% | 1.0E-91 |
| G | SNP (transition) | 11,332 | 11,332 | ORF1ab | A -> G | GTA -> GTG | - | 99.2% | 8.7E-91 |
| T | SNP (transition) | 12,525 | 12,525 | ORF1ab | C -> T | ACA -> ATA | T4087I | 68.6% | 1.2E-63 |
| T | SNP (transition) | 13,019 | 13,019 | ORF1ab | C -> T | CTA -> TTA | - | 31.7% | 1.3E-29 |
| C | SNP (transition) | 13,216 | 13,216 | ORF1ab | T -> C | GAT -> GAC | - | 68.6% | 1.2E-63 |
| T | SNP (transition) | 14,408 | 14,408 | ORF1ab | C -> T | CCT -> CTT | P4715L | 100.0% | 7.6E-93 |
| T | SNP (transition) | 15,24 | 15,24 | ORF1ab | C -> T | AAC -> AAT | - | 93.3% | 1.1E-86 |
| A | SNP (transition) | 15,451 | 15,451 | ORF1ab | G -> A | GGT -> AGT | G5062S | 100.0% | 7.6E-93 |
| T | SNP (transition) | 16,466 | 16,466 | ORF1ab | C -> T | CCA -> CTA | P5400L | 100.0% | 7.6E-93 |
| T | SNP (transition) | 19,22 | 19,22 | ORF1ab | C -> T | GCT -> GTT | A6318V | 98.4% | 3.7E-91 |
| G | SNP (transversion) | 21,618 | 21,618 | S | C -> G | ACA -> AGA | T19R | 100.0% | 1.2E-92 |
| T | SNP (transition) | 21,846 | 21,846 | S | C -> T | ACT -> ATT | T95I | 26.7% | 6.8E-14 |
| A | SNP (transition) | 21,987 | 21,987 | S | G -> A | GGT -> GAT | G142D | 48.3% | 5.4E-27 |
|  | Deletion | 22,029 | 22,034 | S | #NOME? | - | - | 99.8% -> 100.0% | 6.5E-92 |
| G | SNP (transversion) | 22,917 | 22,917 | S | T -> G | CTG -> CGG | L452R | 100.0% | 3.7E-91 |
| A | SNP (transversion) | 22,995 | 22,995 | S | C -> A | ACA -> AAA | T478K | 100.0% | 3.7E-91 |
| G | SNP (transition) | 23,403 | 23,403 | S | A -> G | GAT -> GGT | D614G | 100.0% | 7.6E-93 |
| G | SNP (transversion) | 23,604 | 23,604 | S | C -> G | CCT -> CGT | P681R | 100.0% | 7.6E-93 |
| A | SNP (transition) | 24,41 | 24,41 | S | G -> A | GAT -> AAT | D950N | 96.6% | 1.2E-89 |
| T | SNP (transition) | 25,469 | 25,469 | ORF3a | C -> T | TCA -> TTA | S26L | 100.0% | 7.6E-93 |
| C | SNP (transition) | 26,767 | 26,767 | M | T -> C | ATC -> ACC | I82T | 99.8% | 1.2E-92 |
| C | SNP (transition) | 27,638 | 27,638 | ORF7a | T -> C | GTT -> GCT | V82A | 100.0% | 2.7E-89 |
| C | SNP (transition) | 27,645 | 27,645 | ORF7a | T -> C | CCT -> CCC | - | 48.2% | 1.9E-43 |
| T | SNP (transition) | 27,752 | 27,752 | ORF7a | C -> T | ACA -> ATA | T120I | 100.0% | 4.9E-90 |
| T | SNP (transition) | 27,874 | 27,874 | ORF7b | C -> T | ACT -> ATT | T40I | 99.4% | 2.8E-92 |
| - | Deletion | 28,248 | 28,253 | ORF8 | #NOME? | - | - | 100.0% | 7.6E-93 |
| T | Deletion | 28,27 | 28,271 | Intergenic | TA -> T | - | - | 99.8% | 7.1E-81 |
| G | SNP (transition) | 28,461 | 28,461 | N | A -> G | GAC -> GGC | D63G | 100.0% | 1.5E-91 |
| T | SNP (transversion) | 28,881 | 28,881 | N | G -> T | AGG -> ATG | R203M | 99.8% | 1.0E-91 |
| T | SNP (transversion) | 28,916 | 28,916 | N | G -> T | GGT -> TGT | G215C | 98.6% | 1.3E-90 |
| T | SNP (transversion) | 29,402 | 29,402 | N | G -> T | GAT -> TAT | D377Y | 100.0% | 4.3E-92 |
| T | SNP (transversion) | 29,742 | 29,742 | 3'-UTR | G -> T | - | - | 100.0% | 5.6E-91 |
| A | SNP (transversion) | 29,867 | 29,867 | 3'-UTR | T -> A | - | - | 100.0% | 1.0 |
