# Supplementary material for: Introduction, Dispersal, and Predominance of SARS-CoV-2 Delta Variant in Rio Grande do Sul, Brazil: A Retrospective Analysis
Source: Microorganisms. 2023 Dec 7;11(12):2938. doi: 10.3390/microorganisms11122938 (PMC10745878; doi:10.3390/microorganisms11122938)
Supplement: Supplementary file 1 [file microorganisms-11-02938-s001.zip › Table S6.docx]

Table S6. Characteristic mutations of VOCs Gamma and Delta

| **VOC** | **Gene** | **Mutation** | **Biological change** | **Reference** | **Data set** |
| --- | --- | --- | --- | --- | --- |
| Gamma | ORF1a | S1188L | Unknown |  | Covariants; Oubreak.info; WHO |
| Delta | ORF1a | A1306S | Unknown |  | Covariants; Oubreak.info; WHO |
| Gamma | ORF1a | K1795Q | Unknown |  | Covariants;  Oubreak.info; WHO |
| Delta | ORF1a | P2046L | Unknown |  | Covariants; Oubreak.info; WHO |
| Delta | ORF1a | P2287S | Unknown |  | Covariants; Oubreak.info; WHO |
| Delta | ORF1a | V2930L | Unknown |  | Covariants; Oubreak.info; WHO |
| Delta | ORF1a | T3255I | Unknown |  | Covariants; Oubreak.info; WHO |
| Delta | ORF1a | T3646A | Unknown |  | Covariants; Oubreak.info; WHO |
| Gamma | ORF1a | ∆3675/3677 | Unknown |  | Covariants;  Oubreak.info; WHO |
| Gamma / Delta | ORF1b | P314L | Facilitate viral replication and transcription | [79] | Covariants; Oubreak.info; WHO |
| Delta | ORF1b | G662S | Unknown |  | Covariants; Oubreak.info; WHO |
| Delta | ORF1b | P1000L | Unknown |  | Covariants; Oubreak.info; WHO |
| Delta | ORF1b | A1918V | Unknown |  | Covariants; Oubreak.info |
| Gamma | ORF1b | E1264D | Unknown |  | Covariants; Oubreak.info; WHO |
| Gamma | S | L18F | Enhances an evasive immunological advantage to B-cells responses | [[80]](https://paperpile.com/c/jSXWgn/0DQwk) | Covariants; Oubreak.info; WHO |
| Delta | S | T19R | Reduce the effect of monoclonal antibodies | [[64]](https://paperpile.com/c/jSXWgn/E1EiL) | Covariants; Oubreak.info; WHO |
| Gamma | S | T20N | Unknown |  | Covariants; WHO |
| Gamma | S | P26S | Unknown |  | Covariants;  Oubreak.info; WHO |
| Gamma | S | D138Y | Unknown |  | Covariants;  Oubreak.info; WHO |
| Delta | S | G142D | Associated with higher viral load | [[65]](https://paperpile.com/c/jSXWgn/cbwvZ) | Covariants; |
| Delta | S | E156G | Associated with ∆157/158 and L452R confers resistance to antiviral immunity elicited by vaccination | [81] | Oubreak.info |
| Delta | S | ∆156/157 | Associated with E146G and L452R confers resistance to antiviral immunity elicited by vaccination | [[81]](https://paperpile.com/c/jSXWgn/nnvOz) | Covariants;  Oubreak.info; WHO |
| Delta | S | R158G | Unknown |  | Covariants; WHO |
| Gamma | S | R190S | Unknown |  | Covariants;  Oubreak.info; WHO |
| Gamma | S | K417T | Increases ACE-2 receptor affinity  Resistance to neutralizing antibodies and immune escape  Enhances transmissibility | [82,83,84[]](https://paperpile.com/c/jSXWgn/WdRAV) | Covariants;  Oubreak.info; WHO |
| Delta | S | L452R | Increases interaction between RBD domain and ACE-2 receptor  Improves the capacity to avoid a host’s immune response  Increases transmissibility  In association with T478K stabilizes the RBD-ACE2 complex increasing infectivity  Associated with E156G and L452R confers resistance to antiviral immunity elicited by vaccination | [66,75,81,85,86,87,88] | Covariants;  Oubreak.info; WHO |
| Delta | S | T478K | In association with L452R stabilizes the RBD-ACE2 complex increasing infectivity  Improves host’s immune response escape | [[66,75,85,87]](https://paperpile.com/c/jSXWgn/zABZ9) | Covariants; Oubreak.info; WHO |
| Gamma | S | E484K | Increases ACE-2 receptor affinity  Resistance to neutralizing antibodies  Resistance to monoclonal antibody neutralization  Enhances transmissibility | [[66,75,89,90,91]](https://paperpile.com/c/jSXWgn/YtGlJ) | Covariants; Oubreak.info; WHO |
| Gamma | S | N501Y | Increases replication speed  Increases ACE-2 receptor affinity  Increases virulence and infectivity  Enhances resistance against neutralizing antibodies  May increase viral load | [[66,75,89,90,92,93]](https://paperpile.com/c/jSXWgn/YtGlJ) | Covariants;  Oubreak.info; WHO |
| Gamma / Delta | S | D614G | Increases replication speed  Increases Virulence  Increases cleavage rate at cleavage site S1/S2  Increases susceptibility to monoclonal antibodies and resistance to neutralizing antibodies  Promote the open conformation of RBD domain increasing ACE2 affinity  May increase viral load  Promotes cell entrance | [[66,67,68,69,70,71,](https://paperpile.com/c/jSXWgn/PywD1)  72[,73,74]](https://paperpile.com/c/jSXWgn/PywD1) | Covariants;  Oubreak.info; WHO |
| Gamma | S | H655Y | Associated with changes in antigenicity by conferring escape from human monoclonal antibodies | [[95]](https://paperpile.com/c/jSXWgn/NTXZl) | Covariants;  Oubreak.info; WHO |
| Delta | S | P681R | Located in furin cleavage site S1 /S2 increasing cleavage rate and cell invasion  Increases infectivity and transmissibility | [[66,75,76,77]](https://paperpile.com/c/jSXWgn/jqjT8) | Covariants;  Oubreak.info; WHO |
| Delta | S | D950N | Unknown |  | Covariants; Oubreak.info; WHO |
| Gamma | S | T1027I | Unknown |  | Covariants;  Oubreak.info; WHO |
| Gamma | S | V1176F | Unknown |  | Covariants;  Oubreak.info; WHO |
| Delta | ORF3a | S26L | Unknown |  | Covariants; Oubreak.info; WHO |
| Gamma | ORF3a | S253P | Unknown |  | Covariants; Oubreak.info; WHO |
| Delta | M | I82T | Unknown |  | Covariants;  Oubreak.info; WHO |
| Delta | ORF7a | V82A | May facilitating viral pathogenesis | [[96]](https://paperpile.com/c/jSXWgn/ita1M) | Covariants; Oubreak.info; WHO |
| Delta | ORF7a | T120I | May facilitating viral pathogenesis | [[96]](https://paperpile.com/c/jSXWgn/ita1M) | Covariants; Oubreak.info; WHO |
| Delta | ORF7b | T40I | Unknown |  | Oubreak.info |
| Gamma / Delta | ORF8 | S84L | Unknown |  | Oubreak.info; WHO |
| Gamma | ORF8 | E92K | Unknown |  | Covariants; Oubreak.info; WHO |
| Delta | ORF8 | ∆119/120 | Unknown |  | Covariants;  Oubreak.info; WHO |
| Delta | N | D63G | Unknown |  | Covariants; Oubreak.info; WHO |
| Gamma | N | P80R | Unknown |  | Covariants; Oubreak.info; WHO |
| Gamma / Delta | N | R203K/M | Enhances replication speed, viral fitness and pathogenesis | [[97,98]](https://paperpile.com/c/jSXWgn/Igndj) | Covariants; Oubreak.info; WHO |
| Gamma | N | G204R | Enhances replication speed, viral fitness and pathogenesis | [[98]](https://paperpile.com/c/jSXWgn/tXw9t) | Covariants; Oubreak.info; WHO |
| Delta | N | G215C | Unknown |  | Oubreak.info; WHO |
| Delta | N | D377Y | Unknown |  | Covariants; Oubreak.info; WHO |
| Delta | ORF9b | T60A | Unknown |  | Covariants |
